# A qualitative study exploring the acceptability of the McNulty-Zelen design for randomised controlled trials evaluating educational interventions

**DOI:** 10.1186/s12875-015-0356-0

**Published:** 2015-11-17

**Authors:** Cliodna McNulty, Ellie J. Ricketts, Claire Rugman, Angela Hogan, Andre Charlett, Rona Campbell, on behalf of the CIRT study group

**Affiliations:** Public Health England Primary Care Unit, Microbiology Department, Gloucestershire Royal Hospital, Great Western Road, Gloucester, GL1 3NN UK; Cardiff University, Cardiff, Wales UK; Public Health England Primary Care Unit, Integrated Biobank of Luxembourg, 6, rue Nicolas Ernest Barblé, Luxembourg, L-1210 Luxembourg; Modelling and Economics Department, Public Health England, 61 Colindale Avenue, London, NW9 5EQ UK; Public Health Research, School of Social and Community Medicine, University of Bristol, Canynge Hall, Whiteladies Road, Bristol, BS8 2PR UK

**Keywords:** Education for Health Care Professionals, Clinical trials (epidemiology), Public Health Ethics, Primary Care, Ethics, Screening

## Abstract

**Background:**

Traditional randomised controlled trials evaluating the effect of educational interventions in general practice may produce biased results as participants know they are being evaluated. We aimed to explore the acceptability of a McNulty-Zelen Cluster Randomised Control Trial (CRT) design which conceals from educational participants that they are in a RCT. Consent is obtained from a trusted third party considered appropriate to give consent on participants’ behalf, intervention practice staff then choose whether to attend the offered education as would occur with normal continuing professional development.

**Methods:**

We undertook semi structured telephone interviews in England with 16 general practice (GP) staff involved in a RCT evaluating an educational intervention aimed at increasing chlamydia screening tests in general practice using the McNulty-Zelen design, 4 Primary Care (PC) Research Network officers, 5 Primary Care Trust leads in Public or sexual health, and one Research Ethics committee Chair. Interviews were undertaken by members of the original intervention evaluation McNulty-Zelen design RCT study team. These experienced qualitative interviewers used an agreed semi-structured interview schedule and were careful not to lead the participants. To further mitigate against bias, the data analysis was undertaken by a researcher (CR) not involved in the original RCT.

**Results:**

We reached data saturation and found five main themes;

*Support for the design*: All found the McNulty-Zelen design acceptable because they considered that it generated more reliable evidence of the value of new educational interventions in real life GP settings.

*Lack of familiarity with study design*: The design was novel to all. GP staff likened the evaluation using the McNulty–Zelen design to audit of their activities with feedback, which were to them a daily experience and therefore acceptable.

*Ethical considerations*: Research stakeholders considered the consent procedure should be very clear and that these trial designs should go through at least a proportionate ethical review. GP staff were happy for the PCT leads to give consent on their behalf.

*GP research capacity and trial participation*: GP staff considered the design increased generalisability, as staff who would not normally volunteer to participate in research due to perceived time constraints and paperwork might do so.

*Design* ‘*worth it*’: All interviewees agreed that the advantages of the “more accurate” or “truer” results and information gained about uptake of workshops within Primary Care Trusts (PCTs) outweighed any disadvantages of the consent procedure.

**Discussion:**

Our RCT was evaluating the effect of an educational intervention to increase chlamydia screening tests in general practices where there was routine monitoring of testing rates; our participants may have been less enthusiastic about the design if it had been evaluating a more controversial educational area, or if data monitoring was not routine.

**Implications:**

The McNulty-Zelen design should be considered for the evaluation of educational interventions, but these designs should have clear consent protocols and proportionate ethical review.

**Trial registration:**

The trial was registered on the UK Clinical Research Network Study Portfolio database. UKCRN9722.

**Electronic supplementary material:**

The online version of this article (doi:10.1186/s12875-015-0356-0) contains supplementary material, which is available to authorized users.

## Background

The method used to select participants within a Randomised Controlled Trial (RCT) is a crucial aspect of trial design [1]. In a traditional RCT, individuals are the unit of analysis and are invited to participate and consent; it is at this point randomisation to an intervention occurs. In a standard cluster randomised trial groups of participants (“clusters”), rather than individuals, are invited to participate and consent to take part before randomisation. The consent procedure can lead to loss of eligible clusters, because of low recruitment [2, 3].

**Table:** 

**What this paper adds**	
**What is already known on this subject:**	
Studies have found that Zelen designs increase external and internal validity, but there has been little research into their acceptability with participants who have been involved in Zelen design studies.	
**What this study adds:**	
The study suggests that the McNulty-Zelen design, in which participants are unaware of their participation in a trial where consent is given by a trusted third party, is acceptable to both stakeholders and GP staff in primary care for the evaluation of educational interventions. The McNulty-Zelen design is a promising addition to trial designs for the evaluation of educational interventions; the role of the trusted third party giving consent needs to be clearly defined.	

**Fig. 1:**
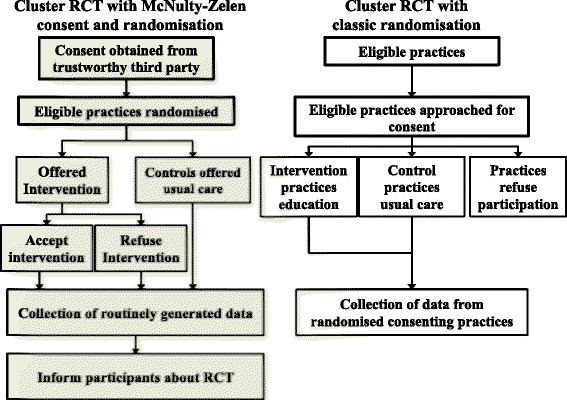
Randomisation and consent with McNulty-Zelen design and usual cluster RCT; detailed legend, comparative processes for traditional cluster RCT and the McNulty-Zelen design used in this study

Selection of only a subgroup of possible participants will affect external validity of a trial; that is the trial’s generalizability (applicability) and relevance to clinical or public health practice [4]. Random assignment to an intervention is considered the gold standard and when properly implemented it eliminates selection bias. Producing unbiased results is particularly challenging in educational interventions involving healthcare staff, as staff in research networks who are the most common participants in research may not be typical, and health care staff interested in the education content are more likely to volunteer. Furthermore, staff who know they are part of an educational evaluation may try harder to change any behaviour they know is being measured [5]. To help overcome these challenges, we recently used a modified Zelen Cluster Randomised Controlled Trial design to evaluate a complex educational intervention aimed at increasing chlamydia screening tests in English general practices [6] (Fig. 1). There are several types of the Zelen design [7]. In the double consent Zelen design all potential participants are first randomised and are then asked to consent; intervention participants can refuse to be involved completely or choose to move over to the control arm. In the single consent Zelen design all participants are randomised to intervention or control, but consent is only sought from the intervention group [8]; the control group receive standard care, and do not know about the intervention or the trial [7]. In our modified Zelen consent variant for educational intervention evaluations, all potential participants are randomised but no consent is obtained from intervention or control groups. Instead consent is obtained from a trusted third party considered appropriate to give consent on participants’ behalf, and then intervention practice staff can choose whether to attend the offered education. This consent is usually obtained before randomisation, so that researchers do not collect any unnecessary information about practices and their staff who will not be involved. The authors have chosen to call this new modified design the McNulty-Zelen design to differentiate it from other modified Zelen designs. As far as we know this McNulty-Zelen Design has only been used by our group [6, 9], and a recent editorial critiquing the study questioned “*was that deception necessary*?” [10] Neither its acceptability, nor that of the Zelen Design, has been evaluated with participants involved in such a study.

We aimed to explore the views of our trial participants, and other stakeholders involved in primary care research, about the acceptability of the McNulty-Zelen design for evaluation of educational interventions. We used the chlamydia RCT as an exemplar.

## Methods

### RCT design

Our RCT using the McNulty-Zelen Design has been previously described [6]. Briefly, 160 general practices were block randomised to the intervention or control. Intervention practices were offered an educational outreach workshop and two further contacts with a chlamydia support worker. The RCT design was discussed and approved by the Warwickshire Research Ethics Committee (REC Ref: 08/H1211/57) and locally approved by Primary Care trusts (PCTs). Informed consent for practice involvement was sought from the Public Health or sexual health leads within each PCT.

The educational intervention took place between April 2010 and April 2011, and in December 2011, after process evaluation interviews had been completed, all randomised surgeries were sent a letter to let them know that their practice had been taking part in a RCT of an intervention aimed at increasing chlamydia screening in general practice, and providing information about their random allocation, the consent procedure, the reason for the trial design and references explaining the Zelen design [7] Additional file 1.

### Qualitative interviews

The interviews were approved in a substantial amendment (May 2011; REC Ref: 08/H1211/167) to the original RCT trial through the Ethics Committee. The qualitative interviews took place between December 2011 and June 2012, 1–6 months after the explanatory letters and one year after intervention completion, but before any publication of trial results.

### Participant selection

#### GP staff

To reduce work overload for the GP staff and any repetition effects arising from being interviewed on two separate occasions, we excluded intervention practices that had taken part in previous process evaluation interviews; these excluded practices did not differ in any way from the Zelen interview practices in terms of practice list size, chlamydia screening rates, deprivation, or training doctors in the practice. The intervention (whether they agreed to workshops or not) and control surgeries were approached in random order. In each selected practice we invited the clinical lead for research or training to participate in the interviews, or to nominate an appropriate individual to participate. From previous experience of time taken to reach data saturation, we aimed to recruit up to 16 GP practice staff.

#### Other stakeholders

We used maximum variation sampling and purposively invited a range of stakeholders involved in coordinating research in Primary Care Research Networks (PCRNs), PCT leads in similar positions to those who were involved in giving consent for our research, and members of research ethics committee to participate. We aimed to interview ten stakeholders.

All potential participants were approached by telephone and those interested in participating were asked to give informed consent by post or email. Participants were not offered any financial incentives.

#### Interview schedule

A semi-structured interview schedule (Additional files 2 and 3) was developed for the telephone interviews by the research team. To ensure understanding of the design, at the start of each interview the researchers gave an oral explanation of the McNulty-Zelen design. The interview schedule then explored using open questions and probing: participants’ thoughts on the benefits and disadvantages of the design, the ethics procedure, how involvement in a traditional RCT design may have influenced their participation or behaviour, and whether use of McNulty-Zelen designs should be encouraged. If participants did not understand the McNulty-Zelen design, this was explained to them in a factual way so that they could answer the later questions on the advantages and disadvantages of the design. GP staff in control or intervention groups were also asked hypothetical questions about their reactions if they had been in the other group, and if they were asked to consent to participate in the RCT. Interviewers were careful to ensure that the participants fully understood the question, and gave further explanation where needed. The stakeholders were also asked about the new National Research Ethics Service (NRES) procedures from 2011, which no longer requires ethical approval for research that only involves NHS staff [11]. GP staff were not asked these questions, as their interviews took place before the study group was aware of the planned change in regulations, and it was not considered appropriate to re-interview them.

#### Interview procedure

Three researchers undertook telephone interviews (ER, CMCN, EO). The interviewers were part of the original RCT team, but did not interview staff they had trained or knew. Furthermore they were trained in qualitative interviewing techniques (through courses and experience). To prevent bias in data collection, interviewers reflected on their interview technique, feelings, reactions and biases during interviews. They then discussed this with other interviewers and the wider research team to optimise their interviewing technique. One pilot interview with a nurse, from one of the randomly selected practices, informed minor changes to the interview schedules. As data collection progressed, interviewers met together and with the wider research team to discuss the emerging themes noted after the interviews, to check if further probing questions were needed and when data saturation had occurred. After about twelve interviews findings were very consistent, and we therefore stopped interviews after completing 16. The formal data analysis below using NVivo was undertaken by one researcher after all data collection was complete. Interviews lasted 15 to 25 min, were recorded digitally, transcribed verbatim by a research assistant (EO) and cross checked by two further researchers (CR and ER) who checked the transcripts while listening to the recordings.

#### Data analysis

Data were anonymised. CR, who was not involved in the RCT, undertook comprehensive analysis of all transcripts. A six-stage Thematic Analysis using an inductive approach was used by the three researchers coding data (CR, CMCN, ER) [12] Additional file 4. 1) Familiarising yourself with the data: First initial notes and ideas were recorded by CR, listening to the audio of each interview when reading and rereading the transcripts. 2) Generating initial codes: QSR NVivo 10 was then used to comb the data line by line for themes, ideas, concepts, terms, phrases and categories, and descriptive codes were generated [13]. 3) Searching for themes: Similarly coded data were collated together and initial ideas were mapped on to thematic models. Researchers analysed answers to the hypothetical questions about their behaviour if they had been in the other arm of the RCT carefully making sure that the participants fully understood the scenario being described. 4) Reviewing themes: two other researchers (ER and CMCN) read and coded 20 % of the transcripts and the codes generated by the additional researchers were reviewed and discussed by three authors (CR, ER, CMcN); there were no significant disagreements about the themes. 5) Defining and naming themes: names for each theme were then agreed and CR revisited the data refining themes. Using the full data set and discussions CR made a Thematic Map. In the thematic map arrows indicate where respondents made links between the themes. Data extracts were selected to illustrate these main thematic findings [12]. 6) Producing the report: the final report was written.

## Results

All practices involved in the original RCT were invited to contact the project manager with any concerns following the information letter sent to them about their random allocation, the consent procedure, the reason for the trial design and references explaining the Zelen design, but none did so.

**Fig. 2:**
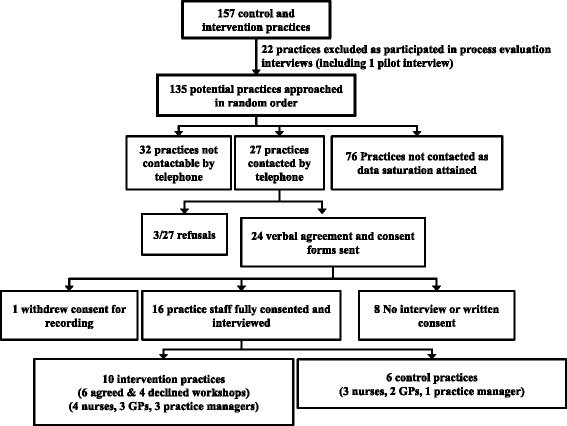
Recruitment of GP staff for interviews; detailed legend, Flowchart depicting process of recruitment of GP staff for interviews

Twenty four of 27 GP practices approached gave verbal approval for interviews. Three practices indicated they did not have time to participate. Eighteen interviews were arranged and written consent was sought. One participant withdrew and one refused consent for transcription. Sixteen interviews were analysed from ten intervention practices (6 who agreed to workshops and 4 who did not; 4 Nurses, 3 GPs, 3 practice managers) and six control practices (3 nurses, 2 GPs, 1 practice manager). Ten of 12 stakeholders approached agreed to participate (4 PCRN officers, 5 PCT leads in Public or sexual health, and a Chair of a Research Ethics committee) (Fig. 2). Initial analysis conducted as interviews were ongoing indicated that data saturation was reached. Later interviews and full analysis of all interviews confirmed this.

**Fig. 3:**
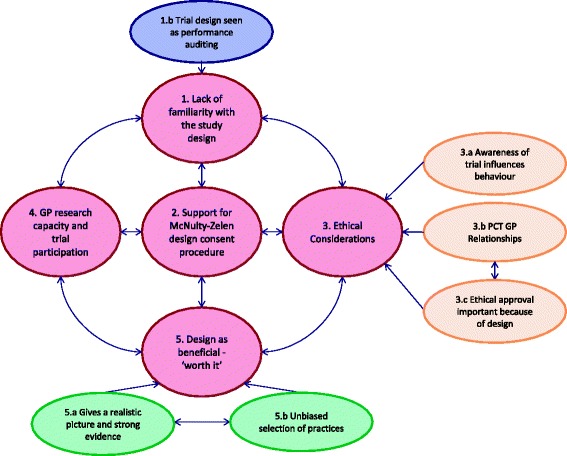
Thematic Map of Main Themes found in qualitative interviews; detailed legend, Map of 5 themes from Thematic Analysis of all interviews

The thematic map illustrates how four themes of initial lack of familiarity with the design, ethical considerations, the design being worth it, and GP research capacity feed in to the final theme; overall support for the McNulty-Zelen design consent procedure (Fig. 3).

**Lack of familiarity with the study design**; Table 1: The theme of lack of familiarity was a fundamental part of the interview results, and none of the GP staff participants reported that they had heard of it prior to receiving information about it from the researchers, several needed reminding at the interview.

*Trial design seen as feedback and monitoring*: The participants, particularly the GP staff, considered the trial design was similar to the way their activities were monitored, and fed back to them by the PCT and others. As GP staff felt their daily work often involved meeting targets this was a familiar concept and made the design very acceptable to them.

**Table 1:** Lack of familiarity with the study design

Lack of familiarity with the design:	
Z8, NURSE: *“I was unsure of what the design was having not heard about the Zelen design before and never been involved in a project that incorporated this type of auditing”*	
Z13, GP: *“I was quite fascinated by it …whether I truly understand it I don’t know but er it’s intriguing”*	
S1, PCRN SENIOR RESEARCH OFFICER: *“initially I thought well that’s something different because obviously normally we would seek interest from the practice and get their consent initially before they took part in a study so this was obviously the opposite way of approaching it. But then reading through it why it was a good way”*	
Trial design seen as monitoring of performance:	
Z13, GP: *“I go into this assuming something like this is going to happen going to look at your figures PCT’s are always looking at your figures”*	
Z14, GP: *“monitoring of our performance is just sort of second nature”*	

**Support for the consent procedure**; Table 2: Most interviewees found the consent procedure acceptable; all GP staff interviewed felt it was appropriate. None of the interviewees saw the need for additional people to be involved in the consent procedure. Any concerns with the consent procedure were about ensuring that an appropriate person was given the responsibility of consenting, rather than about the actual Zelen design or consent procedure itself. The concerns came from five of the stakeholders (none of the GP staff) who were in roles that had been involved in consenting on behalf of practices, but had not actually been involved in the consent procedure as their predecessor had given consent. These stakeholders questioned whether their role as Directors of Public Health gave them the right to consent on behalf of ‘independent GP practices.’ They wanted to see a clear procedure to choose those who gave consent. The one stakeholder who was involved in the consent procedure did not report any issues with the consent. Some stakeholders were worried that the consent procedure may damage relationships between PCTs and GPs (see next theme) and therefore this needed to be treated sensitively. Some interviewees (5 GP staff and 5 stakeholders) speculated that others, rather than themselves, might be apprehensive about this procedure. Others stated that as only anonymised data were collected this made the consent procedure acceptable. GP staff also felt that further written consent from GP practices was unnecessary as both practice managers and GP staff had the choice not to attend the educational workshops, and could refuse any further visits or contacts by the chlamydia coordinator, and they also knew their chlamydia testing rates were already being monitored. The fact that practices and staff in the original trial had to opt in to education was implicitly interpreted as “tacit consent”. However this could not be said for control practices. None of the interviewees saw the need for additional people to be involved in the consent procedure.

**Table 2:** Support for the consent procedure

S3, PUBLIC HEALTH MANAGER AND SEXUAL HEALTH LEAD: *“I don’t see any issue with the consent process that you followed”*	
S10, PCRN SENIOR RESEARCH OFFICER: *“because the data is already there then what you’re doing is scrutinising that and looking at to see whether the level of that data collection has been the quality and levels have increased after the intervention so in that sense I guess it’s less.. of a problem”*	
Z6 “*I can see the rationale behind it* [Zelen design] *and that sounds good.”*	

**Ethical Considerations**; Table 3: The ethical implications of the study were discussed in detail by participants.

*Awareness of trial influences behaviour*: All participants reported that they viewed the trial design as appropriate because awareness of participation in the trial could have of itself influenced their own or others behaviour, which could have biased the trial findings. This was seen as part of human nature, and as such something that could not be avoided, unless the McNulty-Zelen or similar design was used. Some participants noted that the problems associated with this lack of individual informed consent needed to be balanced with bias caused by awareness of the trial. 14 interviewees, both stakeholders and GP staff stressed the importance of informing participants about the design after trial completion, and spoke positively about the explanatory letter about the design researchers had sent to each practice, seeing this as an important part of transparency and trust in the ethical procedure.

*PCT GP Relationships*: Some GPs felt that the relationships between certain PCTs and General Practices were not good, but none had concerns about the PCT sexual health leads consenting on their behalf. In contrast, although the PCRN and PCT stakeholders were happy that the process was ethical, five of the stakeholders were worried that the consent procedure may damage already fragile relationships between GP staff and PCTs. Their anxiety was that GP staff would be offended or upset that PCTs had consented on their behalf, and possibly that GP staff would therefore feel they had not been given a choice. The pre-existing issues between them mentioned by GP staff and stakeholders were a lack of trust and communication which may be exacerbated by the consent procedure.

*Ethical approval important because of design*: Most stakeholders felt there was a need for some sort of ethical approval being sought for this novel design; this was additional to the PCT consent procedure and local approvals that were in place for the trial. There was a strong feeling from stakeholders that there should be a nationally agreed ethical process for trials where consent was not sought, even if the intervention only involved health care staff. This was because of the novel design and the unusual consent procedure that it entailed; some suggested this could involve only a proportionate or chairman’s review.

**Table 3:** Ethical Considerations

*Z2, PRACTICE MANAGER: “where we’re not notified in advance actually is a really good idea it gives you a really true picture of the reality”*	
*S6, ASSISTANT JOINT COMMISSIONING MANAGER: “I just think the perception of some individuals may be that it’s slightly underhand”*	
Awareness of trial influences behaviour:	
*Z15, NURSE: “I think it’s a very good thing if you do research without us knowing about it really because you’re getting a true life situation aren’t you? Actually rather us thinking oh yes we really need to do that, we’d better do that because we’re in a trial.” “Its natural reaction isn’t it to think well we’re doing this therefore we’ve got to perform better at this.”*	
*Z10, GP: “you might feel obliged to increase your screening so I think blindness is useful”*	
*Z9, NURSE: “as far as consent for this goes …if somebody is taking that out of our hands and doing it as a at a PCT level then that’s fine but I think sometimes it’s nice to know that that an annual thing or an on-going thing so we consenting that it’s going to happen but we don’t necessarily need to know when it’s going to happen.”*	
PCT GP Relationships:	
*S4, PUBLIC HEALTH COMMISSIONING MANAGER - SEXUAL HEALTH: “I worry quite a lot that we … are investing quite a lot of work in primary care to make them see that we’re working together and that we can all trust each other, and I just worry that they might get frustrated that were kept out of that decision.”*	
*Z9, NURSE: “Some people might always look on the bleak side, and think you know are they trying to catch us out, you know what are they trying to gain from it?”*	
Ethical approval important because of design:	
*S8, PCRN RESEARCH OFFICER: “Because it’s so novel it’s such a novel design you do need something in place that will ensure GPs that the research was ok so for this type of design. I would actually probably be seeking the ethics for educational research.”*	
*S1, PCRN SENIOR RESEARCH OFFICER: “Approval should still be sought because I think there still needs to be some checks and balances on the study design.”*	
*S5, REC CHAIR: “I think it would be helpful if this sort of [Zelen] design was at least shown to a chair to confirm or make everybody feel comfortable that it really was the sort of Zelen design that could raise no issues.”*	

**GP research capacity and trial participation**; Table 4: Most GP staff reported that their capacity to participate in research was constrained because of time limitations. Many said that if the trial had used a traditional design and consent procedure the real and perceived extra time and paperwork associated with research for them and their practice might have dissuaded them from participating. They reported that the McNulty-Zelen design reduced this research paperwork and therefore allowed more non-research practices to participate in the educational intervention evaluation, so increasing generalizability of results.

**Table 4:** GP research capacity and trial participation

*Z14, GP: “There’s also kind of competing interests of a day to day job that increasingly sort of jam packed. And with the best will in the world you sometimes look at things already sent to them and think oh that looks like a really useful thing to … fill in or contribute to. Actually you may not actually get around to it,- so I suppose this is at least one way of ensuring participation of the people you’re trying to look at”*	
*Z2, PRACTICE MANAGER: “It is possible I’d have chuckled and said oh we haven’t got time to do that because that is very much how that’s my first thought is time. … I think the answer to that would have to be, it depends and I’m sorry it’s not a clear answer but we make a decision at this moment in time based on a whole kind of factors, so at the time when we received the information we would have sat down and decided whether that was the right thing for the practice.”*	
*Z15, NURSE: “I might have said oh no not another trial, I haven’t got time! But in actual fact it didn’t involve us doing anything did it?”*	
*Z12, NURSE: “But whether or not the doctor would have agreed to it would have been [the deciding thing] he more than likely discouraged it because of time, and if he’d have to get involved personally would have probably declined it because of his time”*	

**Design as beneficial; ‘worth it’**; Table 5: This theme was alluded to in a number of ways by most participants. The views expressed were generally that the benefits of the study outweighed the disadvantages and made it worthwhile. Most participants did not perceive there being any disadvantages to using the McNulty-Zelen design.

*Gives a realistic picture and strong evidence*: Almost all participants talked about the trial design being beneficial, as it gave a truer picture or more accurate result, and therefore the evidence was stronger than in a RCT involving research practices.

*Less skewed selection of practices*: That the trial design produced a “less skewed” selection of practices taking part because consent to participation was given by the PCT and not just research practices were involved was seen as an added benefit of this study.

**Table 5:** Design as beneficial; ‘worth it’

*Z12, NURSE: “As long as there’s some useful information that comes from it, I’ve got no personal objections to being part of trial that somebody else has, that the PCT have agreed to.”*	
*S5, REC CHAIR: “I happen to think that the benefits far, far outweigh any of the disadvantages of this particular study”*	
*S2, COMPREHENSIVE LOCAL RESEARCH NETWORK RESEARCH, MANAGEMENT AND GOVERNANCE (RM&G) MANAGER: “They [GP staff] can see reasons for it then I think that would probably justify it.”*	
Gives a realistic picture and strong evidence:	
*Z17, NURSE: “Because it doesn’t give you um a rose coloured view of what’s going on it gives you the true picture.”*	
*S4, PUBLIC HEALTH COMMISSIONING MANAGER - SEXUAL HEALTH: “You should get just a pure result depending on the intervention, yes not based on … any sort of self-selection or wanting to achieve, … a better result because you are in a trial … so I think that is a huge advantage of this [design].”*	
*S6, ASSISTANT JOINT COMMISSIONING MANAGER: “I think … if people don’t know that they’re involved then they carry on with their normal behaviour regardless, and therefore arguably you get a truer picture of .what they do and how they go about doing it, um without behaviour modifications to conform to um any expectations from a study being carried out.”*	
*Z8, NURSE: “The audit is always going to be accurate and valid …because we didn’t know what was going on and we were unaware that we were being visited. I think the information gleaned can then be very accurate.”*	
Less skewed selection of practices:	
*S1, PCRN SENIOR RESEARCH OFFICER: “Because if you did approach the practice then obviously you get those that are interested in and have a particular interest in chlamydia screening or who knew their practice were quite good at, at doing what they’re supposed to be doing, so it could skew the study result. Um so I think it was a good way of um getting a sort of cross section of the practices really, rather than just those who had a particular interest.”*	
*Z6, GP: “It would have been an additional inconvenience if () asked for consent”*	

## Discussion

### Main findings

All participants reported that they felt the McNulty-Zelen design generated stronger and more reliable evidence for the value of implementing a new intervention in a real life setting. All GP staff participants and stakeholders felt overall the consent process was acceptable but some stakeholders queried the procedure for identifying who should be responsible for giving consent on behalf of the GP practices. As the design was so novel and consent was not obtained directly, the stakeholders from research networks and ethics committees suggested that even if the intervention only involved NHS staff these sort of trial designs should go through at least a proportionate review by NRES. GP staff likened the study design to audit and feedback which were common, familiar and acceptable processes [14]. All GP staff reported that as knowledge of the trial would have modified their chlamydia testing behaviour irrespective of whether they were in an intervention or control practice, the main advantage for the design was the production of less biased results. Participants thought that a more generalizable range of practices could offer to participate because of the lower paperwork requirements of this design, and data could be collected on workshop participation rates.

### Strengths and limitations

As far as we know this is the first study exploring the opinions of GP staff or stakeholders actually involved in a Zelen RCT. We consider our findings are transferable to other studies evaluating Public Health or educational interventions in Primary Care across England as we recruited a diverse sample of GP staff from a range of different GP practices and stakeholders. We also interviewed both control and intervention practice staff (including staff in practices who were randomised to the intervention, but did not agree to the chlamydia support worker led workshops). One of the main reasons the participants found the design so acceptable is because they likened the research to performance auditing as they knew that data about their chlamydia screening rates was already being collected. Thus the findings may not be as applicable to educational interventions where routine data is not being routinely collected and monitored. The interviews were by financial necessity undertaken by members of the RCT study team, and therefore this could have introduced some bias into data collection and probing. However the interviewers were as a result familiar with the design and workshop and could probe appropriately, furthermore the interviewers did not deliver the intervention directly to any staff they interviewed, did not know how active the participating surgeries were at chlamydia screening, were experienced in qualitative methods, used the agreed semi-structured interview schedule and were careful not to lead the participants. To further mitigate against bias, the data analysis (except for some double coding) was undertaken by a researcher (CR) who was not involved in the original RCT. We also reached data saturation relatively quickly and later interviews served to confirm earlier results.

Interviews were undertaken by telephone, which aided recruitment of busy GPs and stakeholders, but may have reduced the flow of conversation and shortened the interviews. We only interviewed one of the Public Health leads who gave the original consent for the Zelen design, as half had changed roles in the time since consent was given in 2008. However we can surmise that the other consenting individuals were happy with the design as it was discussed with them before they gave informed consent, and none of the 16 area leads involved in the original study refused consent.

Recall bias may have biased results as the interviews were undertaken one to six months after letters explaining the study were sent to practices. Some participants reported lack of time to read the Zelen design information sent before the interview. Thus to ensure understanding before the interview the researchers gave a neutral, factual explanation of the design; this may have limited the participants responses. This was countered by probing with open questions.

The GP interviews were undertaken before the study team knew that ethical approval would not be needed for studies just involving NHS staff, and therefore did not ask GP staff their views about the new regulations. GP staff may have had a different view about the new regulations, as they reported the importance of saving time and concentrating on PCT priorities and were less concerned about the consent procedure. We thought it was important to report these stakeholder results as they have important implications for future ethical approvals of similar studies.

### Where this research fits in

One qualitative study has explored with parents, whose newborn infants had previously been enrolled in a completed conventional RCT, their views about a hypothetical situation in which the Zelen design may have been used to recruit their child to the same study [15]. Similar to our GP staff, participants in Snowdon’s qualitative study felt that they did not need to give consent for anonymised data that is routinely collected to be monitored [15]. This is also clearly stated in the UK Governance Arrangements [11]. Parents in Snowden’s qualitative study also thought that the Zelen design may undermine trust between the parent and health-care staff [15]. The major disadvantage of Snowdon’s study is that it was discussing a hypothetical study design [15]. Stakeholders in our study, who were also not directly involved in the intervention, in a similar way thought that the Zelen design may undermine trust between the PCT (who gave consent) and GP staff. This was not mentioned as a disadvantage by any of the GP staff themselves, who reported being accustomed to PCTs measuring their performance. In Snowdon’s work with parents, knowledge of the outcomes of the study and their involvement was important and gave participants a sense of contributing to medicine [15]. This is consistent with our findings as all interviewees supported the research team informing the study participants about the McNulty-Zelen study design and results, reporting that this was an important component of the study design.

#### Improved recruitment

In a systematic review the consent procedure and increased demands on clinician’s time during the research period were the most commonly reported clinician related reasons for non-entry of eligible patients into surgical RCTs [4]. Several of our interviewees reported that they may not have participated in a research related educational intervention because of the perceived extra time, clerical work and data collection expectations in research studies. The use of the Zelen design has been used to increase recruitment resulting in a 6-fold increase in recruitment in an arthroscopy study [16]. Our interviewees’ views agreed with a review of 44 studies using the Zelen design [17], in which half stated that they used the design to reduce bias, and is highlighted by the less than 50 % recruitment in several other GP educational cluster randomised trials [5, 18]. The McNulty-Zelen design has the advantage of demonstrating the real acceptance rate of an educational intervention [6]. Our GP staff report that knowing they were in a trial might make them “*try harder*”, is also reflected in an educational study on respiratory tract infections (RTI) in which the intervention GPs diagnosed significantly more RTIs than GPs in the control arm [5].

We suggest that the McNulty-Zelen design and consent process is similar to a cluster randomised trial, if the PCT were considered the cluster and the GP practices the participants [3]. Within a cluster RCT the decision about whether a particular cluster participates in a trial is taken by a “guardian” who usually has the power to “deliver” that cluster [3]. Examples are chief executive of a hospital, or the managing partner of a GP practice; within the McNulty-Zelen design study consent was taken one step further up from the GP’s involved and was given by PCT Public Health or Sexual Health leads. All the GP staff participants were happy with who gave consent, and stakeholders were happy if the process of identifying a suitable individual to consent was clearly explained. Edwards et al. suggest that in cluster randomised trials the guardians’ consent form should clearly set out their duties [3]. We suggest a similar procedure for McNulty-Zelen designs, as this may go some way to allay the fears of stakeholders about the possible risk to the GPs relationship with a service commissioner [19]. McRae et al. also discussed the ethics of lack of consent in cluster randomised trials [20]. We would suggest that the interviewees provided us with enough evidence to indicate that the criteria in the United States Common Rule [21] governing research which permits a research ethics committee to waive the requirements to obtain informed consent were met. These are: (a) “*The research involves no more than minimal risk to the subjects*” (our education was unlikely to lead to any harm); (b) “*The waiver or alteration will not adversely affect the rights and welfare of the subjects*” (participants in our RCT could refuse the education and only routinely collected data was analysed [6]); (c) “*The research could not practicably be carried out without the waiver or alteration*” (informing all practices about the intervention may have reduced recruitment and increased bias [6]); and, (d) “*Whenever appropriate, the subjects will be provided with additional pertinent information after participation*” (practices were given information after the study end) [6]. The NRES does not have similar clear criteria, but does state that “*there is evidence that a universal insistence on consent can undermine research, introducing bias and limiting recruitment”* [22]. NRES goes on to say that “the *legal position is uncertain*” and “*expert advice is needed on when it is appropriate to conduct research without consent.”*

### Conclusions

While a few interviewees indicated the consent process might reduce trust between GP staff and PCT leads, the results indicate promise for the application of the McNulty-Zelen design for the evaluation of educational interventions where routine anonymised outcome data can be collected, although application in other contexts to examine the extent to which this support holds is warranted. However, there are some provisos; primary care stakeholders consider that to reduce any possibility of loss of confidence in the ethical and local review process this sort of study design should be subject to at least proportionate ethical review by NRES. Our RCT was evaluating the effect of an educational intervention to increase chlamydia screening tests in general practices in England where there is a National screening programme and where monitoring of testing rates were routine. Our participants may have been less enthusiastic about the design if it had been undertaken in an area where screening and data monitoring was not routine or we had been evaluating a more controversial educational area. It will be important that the nature of the intervention (whether educational, or how accepted), the exact consent procedure and consent guardian, and data collection are considered when ethics committees approve such designs so that perceived advantages of such a design to increase recruitment and reduce bias do not override the important ethical process and rights of the trial participants. Furthermore, all participants should be informed about the design and results as soon as possible after completion of the intervention evaluation.

### Ethical Approval

The original RCT needs assessment was approved in May 2008 by Warwickshire Research Ethics Committee (REC Ref: 08/H1211/57) with a substantial amendment for the RCT using the Zelen design in March 2010. The interviews for this study were approved in a substantial amendment in May 2011 (REC Ref: 08/H1211/167).

## Additional files

Additional file 1:
**Letter sent to intervention practices after the RCT data collection was complete.** (DOCX 42 kb) Additional file 2:
**Zelen design telephone interview – intervention surgery.** (DOCX 35 kb) Additional file 3:
**Zelen design telephone interview – stakeholders.** (DOCX 34 kb) Additional file 4:
**Stages of thematic analysis by Braun and Clarke.** (DOCX 49 kb)

## Abbreviations

CRT: Cluster Randomised Control Trial
GP: General practice/general practitioner
NHS: National Health Service
NRES: National Research Ethics Service
PCRN: Primary Care Research Networks
PCT: Primary Care Trusts
RCT: Randomised Control Trial
REC: Research Ethics Committee
RTI: Respiratory tract infections

## References

[1] Moher D, Hopewell S, Schulz KF, Montori V, Gøtzsche PC, Devereaux PJ, et al. CONSORT 2010 explanation and elaboration: updated guidelines for reporting parallel group randomised trials. Int J Surg (London, England) [Internet]. Elsevier Ltd; 2012 Jan [cited 2014 Mar 21];10(1):28–55. Available from: http://www.ncbi.nlm.nih.gov/pubmed/22036893 10.1016/j.ijsu.2011.10.001 22036893

[2] Gjelstad S, Høye S, Straand J, Brekke M, Dalen I, Lindbæk M (2013). Improving antibiotic prescribing in acute respiratory tract infections: cluster randomised trial from Norwegian general practice (prescription peer academic detailing (Rx-PAD) study). BMJ.

[3] Edwards SJL, Braunholtz DA, Lilford RJ, Stevens AJ (1999). Ethical issues in the design and conduct of cluster randomised controlled trials. Br Med J.

[4] Abraham NS, Young JM, Solomon MJ (2006). A systematic review of reasons for nonentry of eligible patients into surgical randomized controlled trials. Surgery.

[5] Hutchison D, Styles B (2010). A Guide to Running Randomised Controlled Trials for Educational Researchers.

[6] McNulty C a M, Hogan AH, Ricketts EJ, Wallace L, Oliver I, Campbell R, et al. Increasing chlamydia screening tests in general practice: a modified Zelen prospective Cluster Randomised Controlled Trial evaluating a complex intervention based on the Theory of Planned Behaviour. Sex Transm Inf [Internet]. 2014 May [cited 2014 Apr 15];90(3):188–94. Available from: http://www.ncbi.nlm.nih.gov/pubmed/24005256 10.1136/sextrans-2013-051029 PMC3995257 24005256

[7] Torgerson DJ, Roland M (1998). What is Zelen’s design?. Br Med J (Clinical research ed).

[8] Campbell R, Peters T, Grant C, Quilty B, Dieppe P (2005). Adapting the randomised consent (Zelen) design for trials of behavioural interventions for chronic disease: feasibility study. Jour of Health Serv Res & Pol.

[9] McNulty CA, Thomas M, Bowen J, Buckley C, Charlett A, Gelb D, et al. Interactive workshops increase chlamydia testing in primary care--a controlled study. Fam Prac [Internet]. 2008 Aug [cited 2013 Oct 23];25(4):279–86. Available from: http://www.ncbi.nlm.nih.gov/pubmed/18579709 10.1093/fampra/cmn032 18579709

[10] Miller WC, Nguyen NL. Relative or absolute? A significant intervention for chlamydia screening with small absolute benefit. Sex Transm Infect [Internet]. 2014 May [cited 2014 Apr 22];90(3):172–3. Available from: http://www.ncbi.nlm.nih.gov/pubmed/24719029 10.1136/sextrans-2013-051426 PMC4526139 24719029

[11] DH Research and Development Directorate (England); National Institute for Social Care and Health Research (Wales); Chief Scientist Office (Scotland); R&D Division, Public Health Agency (Northern Ireland). Governance arrangements for research ethics committees: a harmonised edition. May 2011

[12] Braun V, Clarke V (2006). Using thematic analysis in psychology. Qual Res Psychol.

[13] Taylor C, Gibbs GR. “What is Qualitative Data Analysis (QDA)?”, Online QDA Web Site, 2010. http://onlineqda.hud.ac.uk/Intro_QDA/what_is_qda.phpAccessed 30.10.15

[14] Ashcroft DM, Valderas JM, Doran T (2014). Withdrawing performance indicators: retrospective analysis of general practice performance under UK. BMJ.

[15] Snowdon C, Elbourne D, Garcia J. Zelen randomization: attitudes of parents participating in a neonatal clinical trial. Control Clin Trials [Internet]. 1999 Apr;20(2):149–71. Available from: http://www.ncbi.nlm.nih.gov/pubmed/10227415 10.1016/s0197-2456(98)00049-x 10227415

[16] Chang RW, Falconer J, Stulberg SD, Arnold WJ, Dyer AR (1990). Prerandomization: an alternative to classic randomization. The effects on recruitment in a controlled trial of arthroscopy for osteoarthrosis of the knee. J Bone Joint Surg Am.

[17] Adamson J, Cockayne S, Puffer S, Torgerson DJ (2006). Review of randomised trials using the post-randomised consent (Zelen’s) design. Contemp Clin Trials.

[18] Rognstad S, Brekke M, Fetveit A, Dalen I, Straand J (2013). Prescription peer academic detailing to reduce inappropriate prescribing for older patients: a cluster randomised controlled trial. Brit J of Gen Prac [Internet].

[19] Kirkwood BR, Morrow RH (2011). Community-based intervention trials. J Biosoc Sci.

[20] McRae AD, Weijer C, Binik A, Grimshaw JM, Boruch R, Brehaut JC, et al. When is informed consent required in cluster randomized trials in health research? Trials [Internet]. BioMed Central Ltd; 2011 Jan [cited 2014 Feb 4];12(1):202. Available from: http://www.pubmedcentral.nih.gov/articlerender.fcgi?artid=3184061&tool=pmcentrez&rendertype=abstract 10.1186/1745-6215-12-202 PMC3184061 21906277

[21] Department of Health and Human Services. Title 45, Public Welfare, Part 46, Protection of Human Subjects 2009 http://www.hhs.gov/ohrp/policy/ohrpregulations.pdfAccessed 30.10.15

[22] National Research Ethics Service Information Sheets Consent Forms Guidance for Researchers & Reviewers 2011(March). [Accessed April 2014] from http://www.hra.nhs.uk/documents/2013/09/information-sheet-and-consent-form-guidance.pdf

