# Supplementary material for: A qualitative study exploring the acceptability of the McNulty-Zelen design for randomised controlled trials evaluating educational interventions
Source: BMC Fam Pract. 2015 Nov 17;16:169. doi: 10.1186/s12875-015-0356-0 (PMC4647292; doi:10.1186/s12875-015-0356-0)
Supplement: Additional file 1: — Letter sent to intervention practices after the RCT data collection was complete. (DOCX 42 kb) [file 12875_2015_356_MOESM1_ESM.docx]

**Additional file 1: Letter sent to intervention practices after the RCT data collection was complete**

Dear

Health Protection Agency

Chlamydia Support Team

Microbiology Department

FREEPOST NAT13026
Gloucester
GL1 3BR

email: [ellie.ricketts@hpa.org.uk](mailto:ellie.ricketts@hpa.org.uk)

**Re: Chlamydia Screening Study in SW England (REC No. 08/H1211/167)**

This letter is to let you know that your surgery has been taking part in an intervention trial aimed at increasing chlamydia screening in general practice.

During the past year a chlamydia support worker from the HPA has been contacting you on a regular basis; this was part of a trial to encourage increased chlamydia screening. The HPA received ethical approval to undertake a randomized controlled trial with GP practices across the South West of England to establish whether an evidence-based intervention could increase the number of chlamydia screens undertaken at each surgery and, furthermore, whether a higher number of positive screens were detected. The trial was also approved locally by the R&D coordinator and sexual health lead within each PCT.

A Zelen design was approved for this study. In trials of educational interventions it is often the case that enthusiastic practices with an interest in the subject area volunteer to take part, which may lead to exaggeration of any positive outcomes. A better indication of how effective an intervention is can be obtained if participants do not know they are part of an intervention and this is called a modified Zelen design^^[[1]](#footnote-2)^^. See enclosed. This meant that GP surgeries were selected randomly and allocated to one of the arms of the study (either intervention or control) and they did not know they were participating in a research trial. This was in order to ensure a lack of bias in chlamydia screening behaviour by surgery staff and to be able to measure the effect of the intervention as accurately as possible. Your surgery was randomised to the intervention group. This means that we provided you with extra support above that provided by the National Chlamydia Screening Programme team.

If you would like any further information please email Ellie Ricketts, Project Manager [ellie.ricketts@hpa.org.uk](mailto:ellie.ricketts@hpa.org.uk)

Yours sincerely

**Dr Cliodna McNulty**

**Head, HPA Primary Care Unit**

*BMJ* 1998; 316 : 606 (Published 21 February 1998)

**What is Zelen's design?**

[**David J Torgerson**](http://www.bmj.com/search?author1=David+J+Torgerson&sortspec=date&submit=Submit)**, senior research fellow**[^a^](http://www.bmj.com/content/316/7131/606.full#aff-1)**,** [**Martin Roland**](http://www.bmj.com/search?author1=Martin+Roland&sortspec=date&submit=Submit)**, director of research and development**[^b^](http://www.bmj.com/content/316/7131/606.full#aff-2)

1. *^a^ National Primary Care Research and Development Centre, Centre for Health Economics, University of York, York YO1 5DD*
2. *^b^ National Primary Care Research and Development Centre, University of Manchester, Manchester M13 9PL*

When patients do not receive their preferred treatment in randomised trials there may be difficulties with patient recruitment and scientific problems with bias.[^1^](http://www.bmj.com/content/316/7131/606.full#ref-1) For example, bias may occur when patients are aware of a new treatment not available to them and comply poorly with the standard treatment.

Zelen's design can address these difficulties [^2^](http://www.bmj.com/content/316/7131/606.full#ref-2) [^3^](http://www.bmj.com/content/316/7131/606.full#ref-3) by randomising patients *before* consent to participate has been sought. Two types of the design exist: double and single consent. In the double consent version patients are initially offered the treatment to which they were randomised; however, if they decline the randomised treatment, they can then be offered alternative therapies—including the experimental treatment. In the single consent version only patients offered the experimental treatment are told there is an alternative treatment (the control) available. Patients randomised to the control treatment are not allowed the experimental treatment (although they are given unhindered access to any usual treatment facilities). Analysis is undertaken with patients retaining their original assignment.

Zelen's design has been much discussed and for most therapeutic trials is probably unethical. Occasionally, however, it has been chosen on ethical grounds. For example, in a trial of extracorporeal membrane oxygenation for infants with pulmonary hypertension Zelen's design was used as it was considered preferable not to raise false hopes among half the parents that there was a novel treatment available for their child only to have it denied them through the randomisation.[^4^](http://www.bmj.com/content/316/7131/606.full#ref-4)

Zelen's design may be particularly useful for evaluating population based interventions such as screening, where it is important to estimate the effects on a whole population. However, if the presence of the trial is known to the non-screened group this may artificially induce changes in that group which may influence the results (a Hawthorne effect). For example, in a randomised trial of bone density screening[^5^](http://www.bmj.com/content/316/7131/606.full#ref-5) the non-screened group were not contacted at baseline as this might have artificially increased their use of hormone replacement therapy. Had the trialists not used Zelen's design the investigators could not have been sure of the full unbiased impact of screening on uptake of hormone replacement therapy.

If bias due to patients knowing they are in the “usual care” group is to be avoided patients usually need to be followed up for key events at a distance so as not to alert them to the study. For example, in a randomised trial of colorectal cancer screening cancer events for both groups of patients were ascertained through medical records and a cancer registry.[^6^](http://www.bmj.com/content/316/7131/606.full#ref-6) By using Zelen's design in screening trials it is possible to achieve more accurate estimates of population outcomes such as cancer reduction [^6^](http://www.bmj.com/content/316/7131/606.full#ref-6) [^7^](http://www.bmj.com/content/316/7131/606.full#ref-7) compared with the conventional trial designs.

There are obvious ethical problems in using Zelen's design to randomise patients without their consent[^8^](http://www.bmj.com/content/316/7131/606.full#ref-8) (though treatment consent is always sought). For some interventions, however, such as screening, this may be the only practical design. For example, if all patients in the colorectal cancer screening trials had been screened but only a random half had been offered intervention, there would have been an ethical dilemma of not offering further investigation and treatment to control patients who appeared to be at high risk.

Zelen's design can have other disadvantages. If the trial requires intrusive data collection or monitoring then Zelen's design as control patients will be aware of the study. Given that intrusive data collection is not feasible, it may not be possible to use restrictive inclusive or exclusive patient recruitment criteria. Furthermore, if many patients refuse their original treatment, this will lead to a reduction in study power. Both these factors will lead to the need for a large sample size.[^9^](http://www.bmj.com/content/316/7131/606.full#ref-9)

**References**

1. Torgerson DJ,Sibbald B *. What is a patient preference design? BMJ 1998; 316: 360*
2. Zelen M *. A new design for randomized clinical trials. N Engl J Med 1979;300:1242-1245*
3. Zelen M *. Randomized consent designs for clinical trials: An update. Stats in Med 1990;9:645-656.*
4. O'Rourke PP,Crone RK, Vacanti JP, Ware JH,Lillehli CW, Parad RB, et al*. Extracorporeal membrane oxygenation and conventional medical therapy in neonates with persistent pulmonary hypertension of the newborn: A prospective randomized study. Pediatrics 1989;84:957-963*
5. Torgerson DJ, Thomas RE, Campbell MK, Reid DM *Randomised trial of osteoporosis screening: HRT uptake and quality of life results. Arch Intern Med 1997;157:2121-2125*
6. Hardcastle JD, Chamberlain JO, Robinson MHE, Moss SM, Amar SS, Balfour TW et al*. Randomised controlled trial of faecal-occult-blood screening for colorectal cancer. Lancet 1996;348:1472-1477*
7. Kronborg O, Fenger C, Olsen J, Jorgensen OD, Sondergaard O *Randomised study of screening for colorectal cancer by faecal occult blood test. Lancet 1996;348:1467-1471*
8. Smith R *. Informed consent: the intricacies. BMJ 1997;314:1059-1060*
9. Altman DG, Whitehead J, Parmar MKB, Stenning SP, Fayers PM, Machin D  *Randomised consent designs in cancer clinical trials. Eur J Cancer 1995; 31A: 1934-1944*

1. A pure Zelen design means that the participants in the intervention arm are consented to the trial once randomization has taken place. Consent from participants is not sought in a modified design. [↑](#footnote-ref-2)
