# Supplementary material for: A qualitative study exploring the acceptability of the McNulty-Zelen design for randomised controlled trials evaluating educational interventions
Source: BMC Fam Pract. 2015 Nov 17;16:169. doi: 10.1186/s12875-015-0356-0 (PMC4647292; doi:10.1186/s12875-015-0356-0)
Supplement: Additional file 2: — Zelen design telephone interview – intervention surgery. (DOCX 35 kb) [file 12875_2015_356_MOESM2_ESM.docx]

**Additional file 2: Zelen design telephone interview – intervention surgery**

Do you understand the Zelen design?

**Thoughts and feelings on Zelen design**

1 What were your first thoughts on learning about the design of this trial?

2 Does your surgery support research in general by participating in studies or undertaking your own research?

3 How does your surgery encourage continuing professional development of its staff?

4 Were you aware at any point that you were participating in a research trial? Yes/No

4a If yes, why was that?

5 What do you think are the benefits and disadvantages of this design to evaluation public health initiatives in primary care?

6 Consent for this trial was given at PCT level and practices did not consent to take part. What are your thoughts about the consent procedure in this study?

7 Do you think this sort of study should be encouraged?

7a If Yes: Why?

7b If No: How do you think we should evaluate public health interventions and keep selection bias to a minimum?

8. Do you think that lack of consent to take part is an issue if only data that is routinely collected and monitored is used?

**9. *If intervention surgery:***

9a What would your response have been had you been asked for consent to participate in the trial?

9b Do you think you would have taken part?

9c If you knew you were part of a trial do you think it would have influenced your behaviour? What about other staff members in the practice?

[pause]

9d I want you now to consider your reaction if you had been a control surgery. How do you imagine you would have felt if you had not had the opportunity to receive the structured intervention?

10 Only *control surgery questions*

10a What would your response have been had you been asked for consent to participate in the trial?

10b Do you think you would have taken part?

10c If you knew you were part of a trial do you think it would have influenced your behaviour? What about other staff members in the practice?

[pause]

10d I want you now to consider your reaction if you had been an intervention surgery. How would you have felt if you had been offered the opportunity to receive the structured intervention without the knowledge that it was a trial?

**Support for Zelen design**

11 Overall, do you support the method we used (the Zelen design) of recruiting to this trial or not?

11a Why?
