# Supplementary material for: A qualitative study exploring the acceptability of the McNulty-Zelen design for randomised controlled trials evaluating educational interventions
Source: BMC Fam Pract. 2015 Nov 17;16:169. doi: 10.1186/s12875-015-0356-0 (PMC4647292; doi:10.1186/s12875-015-0356-0)
Supplement: Additional file 3: — Zelen design telephone interview – stakeholders. (DOCX 34 kb) [file 12875_2015_356_MOESM3_ESM.docx]

**Additional file 3: Zelen design telephone interview – stakeholders**

**Check comprehension of Zelen design**

Do you understand the Zelen design?

**Thoughts and feelings on Zelen design**

1. What were your first thoughts on learning about the design of this trial?
2. What do you think are the benefits and disadvantages of this design to evaluation public health initiatives in primary care?
3. Consent for this trial was given at PCT level and practices did not consent to take part. What are your thoughts about the consent procedure in this study? (Do you think that lack of consent to take part is an issue if only data that is routinely collected and monitored is used?)
4. Do you think anyone else should have been involved in the consent procedure?
5. Do you think this sort of study should be encouraged?
6. If Yes: Why?
7. If No: How do you think we should evaluate public health interventions and keep selection bias to a minimum?

**Support for Zelen design**

1. Overall, do you support the method we used (the Zelen design) of recruiting to this trial or not?
2. Why?

**NRES in the future**

1. The NRES no longer needs ethical approval for studies just involving health care staff. What are your views about not seeking NRES approval for a Zelen Design Study only involving health care staff?
