# Supplementary material for: A qualitative study exploring the acceptability of the McNulty-Zelen design for randomised controlled trials evaluating educational interventions
Source: BMC Fam Pract. 2015 Nov 17;16:169. doi: 10.1186/s12875-015-0356-0 (PMC4647292; doi:10.1186/s12875-015-0356-0)
Supplement: Additional file 4: — Stages of thematic analysis by Braun and Clarke. (DOCX 49 kb) [file 12875_2015_356_MOESM4_ESM.docx]

**Additional file 4: Stages of thematic analysis by Braun and Clarke^12^**
